# 4D-CT-based motion correction of PET images using 3D iterative deconvolution

**DOI:** 10.18632/oncotarget.26862

**Published:** 2019-04-26

**Authors:** Lena Thomas, Thomas Schultz, Vesna Prokic, Matthias Guckenberger, Stephanie Tanadini-Lang, Melanie Hohberg, Markus Wild, Alexander Drzezga, Ralph A. Bundschuh

**Affiliations:** ^1^ Klinik und Poliklinik für Nuklearmedizin, Universitaetsklinikum Bonn, Bonn, Germany; ^2^ B-IT and Department of Computer Science, Universitaet Bonn, Bonn, Germany; ^3^ University Koblenz-Landau, Department of Physics, Koblenz, Germany; ^4^ University of Applied Sciences Koblenz, Koblenz, Germany; ^5^ Department of Radiation Oncology, University Hospital Zurich, Zurich, Switzerland; ^6^ Department of Nuclear Medicine Universitaetsklinikum Köln, Cologne, Germany

**Keywords:** PET/CT, motion correction, deblurring

## Abstract

**Objectives:**

Positron emission tomography acquisition takes several minutes representing an image averaged over multiple breathing cycles. Therefore, in areas influenced by respiratory movement, PET-positive lesions occur larger, but less intensive than they actually are, resulting in false quantitative assessment. We developed a motion-correction algorithm based on 4D-CT without the need to adapt PET-acquisition.

**Methods:**

The algorithm is based on a full 3D iterative Richardson-Lucy-Deconvolution using a point-spread-function constructed using the motion information obtained from the 4D-CT. In a motion phantom study (3 different hot spheres in background activity), optimal parameters for the algorithm in terms of number of iterations and start image were estimated. Finally, the correction method was applied to 3 patient data sets. In phantom and patient data sets lesions were delineated and compared between motion corrected and uncorrected images for activity uptake and volume.

**Results:**

Phantom studies showed best results for motion correction after 6 deconvolution steps or higher. In phantom studies, lesion volume improved up to 23% for the largest, 43% for the medium and 49% for the smallest sphere due to the correction algorithm. In patient data the correction resulted in a significant reduction of the tumor volume up to 33.3 % and an increase of the maximum and mean uptake of the lesion up to 62.1 and 19.8 % respectively.

**Conclusion:**

In conclusion, the proposed motion correction method showed good results in phantom data and a promising reduction of detected lesion volume and a consequently increasing activity uptake in three patients with lung lesions.

## INTRODUCTION

Positron emission tomography (PET), especially in combination with computed tomography (CT) is gaining more and more influence in the diagnosis, staging, therapy planning and treatment response control in cancer patients [1–4]. As image acquisition takes several minutes PET is sensitive to movement of the patient, especially for periodic motion as breathing. In the lung respiratory movement of up to 24 mm was reported, especially in the lower lobes [5]. Such movement can result in blurred images with lesions appearing larger but less intensive than they actually are. Especially when using quantitative PET features as for therapy response assessment [6] or radiation therapy treatment planning. An additional problem appears in hybrid PET/CT, as a CT is a short ‘snapshot’ image acquisition, sometimes in breath-hold technique, while PET represents an averaged image over several minutes. In the worst case, this causes a misalignment between PET and CT data, which is increased due to patient motion. [7]

Different options have been proposed to overcome this issue. Respiratory gating (4D-CT or 4D-PET) is the most widely distributed and is meanwhile implemented in all commercial PET systems. This method is based on image sorting, providing different images for the different breathing states of the patient and can therefore lead to significant improvements in lesion quantification [1]. But it requires external devices which need to be attached to the patient such as infrared markers or pressure sensitive belts. Additionally, scan time needs to be increased as for each image only a part of the acquired data is used for reconstruction [8–14].

More sophisticated approaches for motion correction were developed over the past years. Some of them correct during the reconstruction process, others using a line of response correction, or using 4D-PET acquisition. For the first two approaches to reconstruction, individual information about the scanner as detector geometry is required. In contrast, corrections in the image space do not require information about the hardware as the are based on the reconstructed images [15, 16]. However, often dynamic PET-imaging as list-mode-acquisition is necessary to include timing information.

Deconvolution techniques have been widely applied in optical analysis and video sequence processing to restore the sharpness of images [17–20]. The process of deconvolution is an inverse filtering process, in which the effects of convolution by a point spread function (PSF) that resulted in the blur, are to be inverted [20]. But other than in a filtering process there are several issues that need to be cautiously addressed to achieve a high-quality deconvolution.

Direct inversion using Fourier-based methods would lead to amplification of noise and degradation in the restored image [19, 20]. Therefore, several regularized solutions have been proposed. A frequently used approach is the Wiener filter [19]. However, the regularization parameter and the power spectrum of noise must be estimated. In addition, negative values may appear in the solution. A better alternative is to use methods with non-negativity constraints such as the minimum residual norm with steepest descent, or the expectation maximization method (EM) [18, 21]. The latter offers a more flexible framework and was used in the study. The EM-based estimate of the estimated unblurred image f after the k-th iteration is given by [21]. This basic iteration is also known as the Richardson-Lucy iteration and could be derived from the maximum likelihood of a Poisson distribution. Accurate implementation of the Richardson-Lucy deconvolution requires complete knowledge of the PSF, which is not always available. This Deconvolution method has been utilized successfully to improve the quality of medical images such as spiral CT acquisition or recently in small animal PET studies [22], where a Gaussian kernel was used to model scattering. If the PSF is not known, blind deconvolution approaches can be used [22, 23].

In this study, we developed a motion-correction algorithm, which derives the motion information from 4D-CT data, often acquired in clinical workflows for radiation therapy treatment planning in the lung or upper abdominal organs. The motion information is then used to construct a 3D PSF to deconvolve the motion image using iterative Richardson-Lucy algorithm.

## RESULTS

### Phantom study

**Figure 1:**
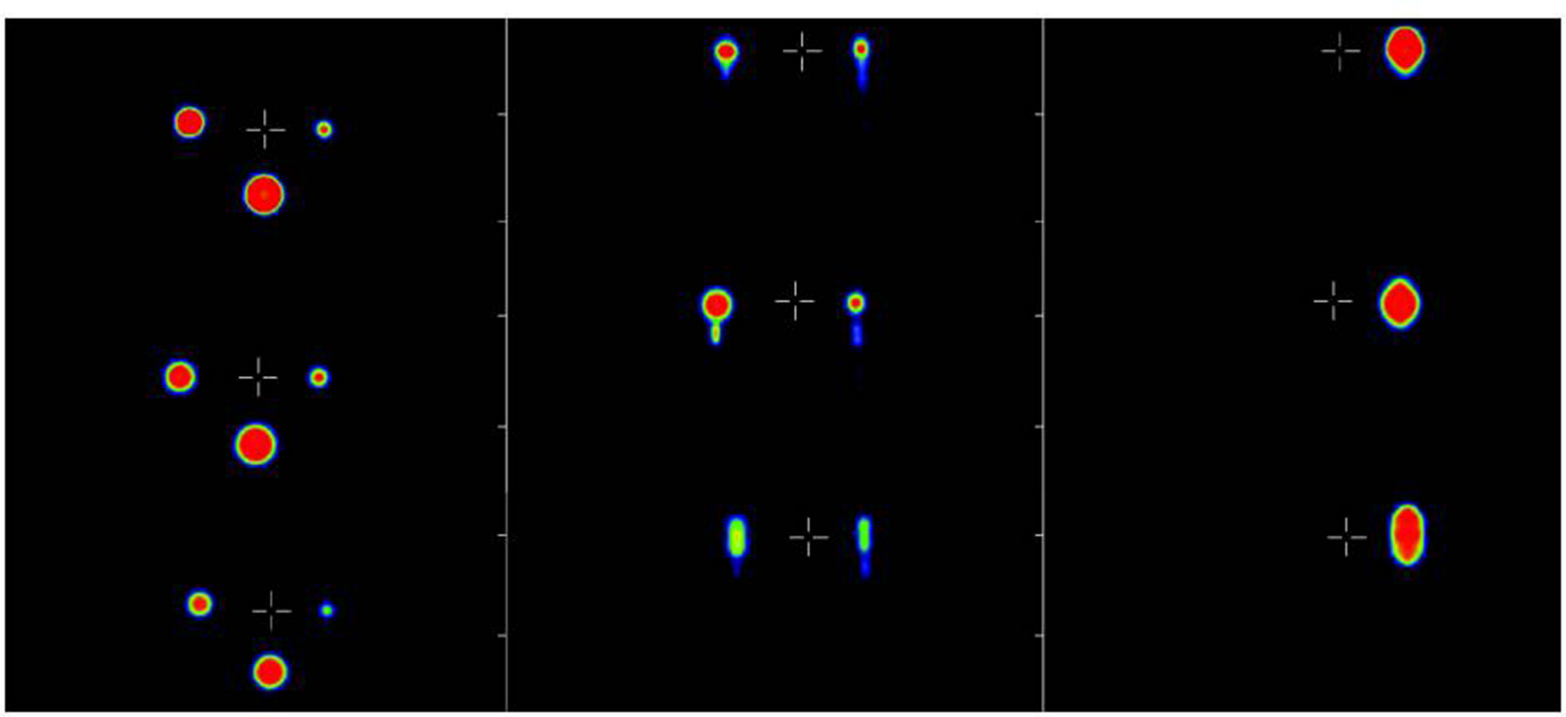
Comparison of the motion phantom with movement (bottom), without movement (top), and after the correction was performed (middle), shown in three different planes (from left to right: axial, coronal and sagittal)

**Figure 2:**
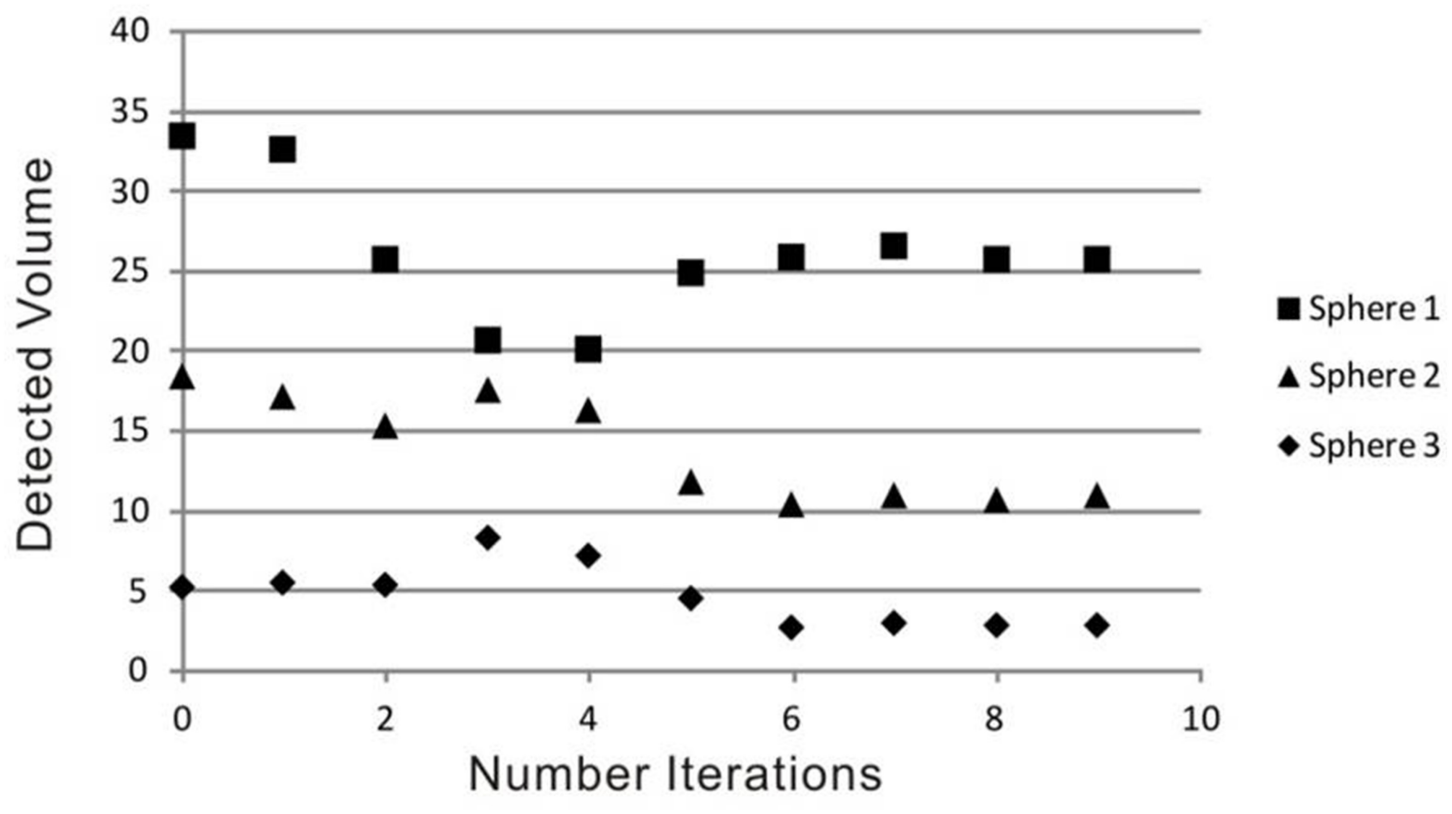
Detected volume in the phantom spheres depending on number of iterations in the deconvolution step (the real volume of the spheres is 26, 12, and 3 ml).

In comparison with the static PET-phantom acquisition (without movement) the results after 6 deconvolution steps or higher showed the best conformity. We found no significant differences between the results using different start images. So, for final analysis the blurred (uncorrected) PET image was used as start image together with 6 iterations steps for the deconvolution. In phantom studies, lesion volume improved up to 23% for the largest, 43% for the medium and 49% for the smallest sphere due to the correction algorithm, a comparison between the static phantom and the corrected data set can be seen in Figure 1. In Figure 2 the detected volume of the three different spheres depending on the number of iterations is shown, for a number of six or more iteration the result is becoming stable. In case of six iterations the detected volume of the spheres improved from 33.4, 18.3, and 5.2 ml to 25.8, 10.4, and 2.6 ml while the real volume was 26, 12, and 3 ml.

**Figure 3:**
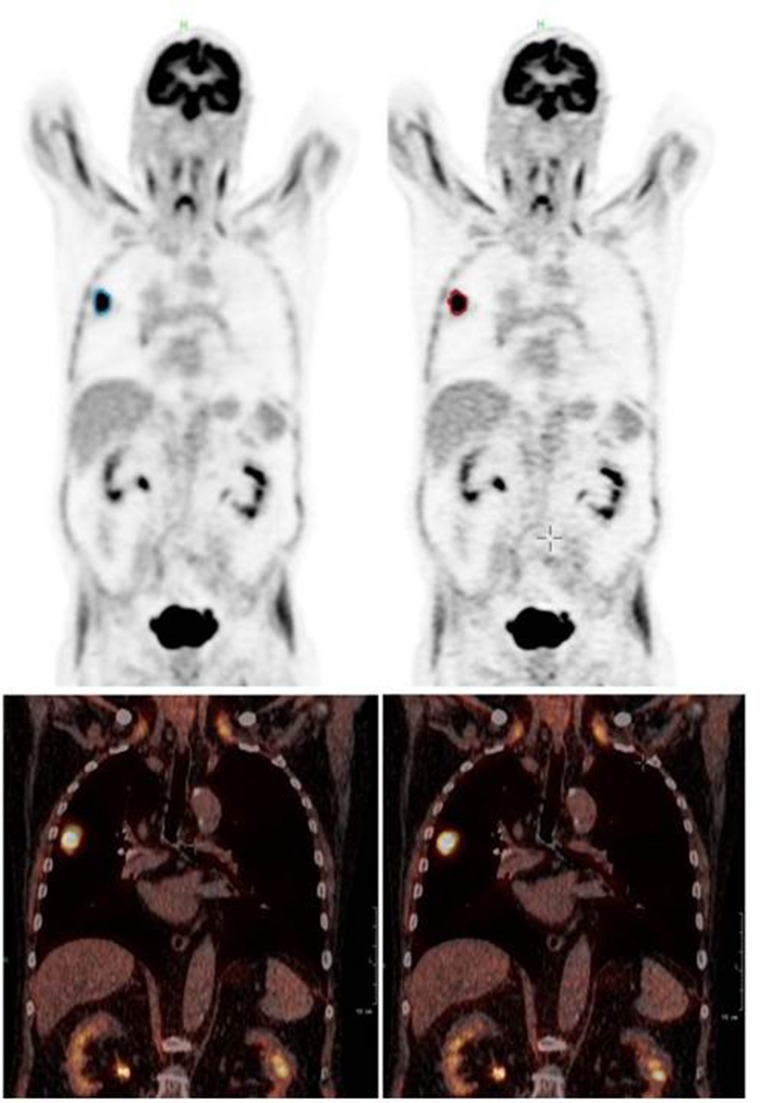
Patient example (Patient 1): coronal PET (top) and fused PET/CT (bottom) of the uncorrected (left) and corrected (right) data set Especially in the fused data set the tumor can be seen less smeared and better fitting to the CT image.

In patient data the detected lung lesion volume decreased between 5.6 and 15.6 % when using just the PSF in cranio-caudal direction and between 25.2 and 33.3 % when using the full 3D PSF for motion correction. Consequently, maximum uptake in the lesion showed an increase between 43.4 and 62.1 % and the mean uptake between 12.0 and 19.8 % when using the full 3D PSF. The details for each individual patient can be found in Table 1, an example can be seen in Figure 3.

**Table 1:** Lesion volume and maximum and mean uptake of the lung lesions of three patients detected in the uncorrected images (uncorr), in the images corrected with a PSF just in cranio-caudal-direction (corr 1D) and in the images corrected using the full 3D PSF (corr 3D).

Patient	volume [ml]			maximum uptake			mean uptake		
	uncorr	corr 1D	corr 3D	uncorr	corr 1D	corr 3D	uncorr	corr 1D	corr 3D
1	40.5	34.2 **(15.6 %)**	30.3 **(25.2 %)**	22.6	24.9 **(10.3 %)**	32.4 **(43.4 %)**	10.0	10.8 **(8.0 %)**	11.2 **(12.0 %)**
2	9.1	8.1 **(11.0 %)**	6.3 **(30.8 %)**	12.5	13.6 **(8.9 %)**	18.9 **(51.2 %)**	7.6	8.2 **(7.9 %)**	8.9 **(17.1 %)**
3	1.8	1.7 **(5.6 %)**	1.2 **(33.3 %)**	16.1	20.9 **(29.8 %)**	26.1 **(62.1 %)**	12.6	13.6 **(7.9 %)**	15.1 **(19.8 %)**

The relative differences can be found in brackets and bold after the values for the corrected data.

## DISCUSSION AND CONCLUSION

Respiratory motion degrades quantitative assessment of lesion volume and activity concentration in PET/CT data of tumors in the lung or the upper abdomen. While many approaches for motion correction are available as discussed in the introduction, the presented algorithm has some essential differences. This algorithm is using clinical 3D-PET images, so it can be applied to all images independent of the scanner type. No dynamic PET scan or special acquisition protocols, or external sensors fixed to the patient, are necessary, as all steps of the algorithm are performed in the image space using reconstructed PET data. The motion vector is obtained by 4D-CT images which are performed often in case of radiation therapy treatment planning. However, motion can also be extracted from other sources, e.g. MR or just from a two phase CT (min and max inhale) or tracked by infrared cameras and markers. Also, movement from sources other than respiration can be corrected as far as motion information is available. Furthermore, the presented motion correction approach can be applied to any (molecular) image, as long as the motion information is given and it is not limited to PET, it also can be applied to single photon emission tomography images. This may even be through for small animal PET imaging as long as the motion vector can be assessed by 4D CT or MRI imaging or any other means of measurement.

The presented CT-based motion correction method showed promising results in phantom and patient data. The real volume of the spheres placed in the phantom could be restored very well. In patient data we found significant reduction of lesion volume up to 33.3 % and consequently an increase in activity uptake of up to 62.1 %. We could also show that the deconvolution using the full 3D PSF showed better reduction in lesion volume and a larger increase in activity uptake of the lesions as the PSF just based on the cranio-caudal movement. This effect was especially seen in patient 2 and 3, in who a relevant movement in the anterior-posterior direction was detected. Therefore, we conclude that a PSF based on the full 3D movement should be applied.

However, a drawback of the method is that a 4D- or cine CT is necessary, as long as no other method of motion detection is used, which means additional dose for the patient. Just two low-dose CTs are possible as well, one taken in end-expiration, the other in end-inspiration. Using actual low-dose protocols, the additional dose will be minimal. Finally, in case PET is used for radiation treatment planning, where precise quantification is of special importance, often a 4D-CT is performed anyway.

In conclusion, the proposed motion correction method showed good results in phantom data and a promising reduction of detected lesion volume and a consequently increasing activity uptake in three patients with lung lesions.

## MATERIALS AND METHODS

This algorithm is using the high spatial and temporal resolution CT data from a hybrid PET/CT for motion registration in 3D to correct the low spatial resolution PET data. The basic principle of this approach was already used by Ben-Ezra et al [24], using a camera hybrid system to detect motion. The design uses a rigid rig of two cameras: a high-resolution camera as the primary detector and a low-resolution video camera as the secondary detector, which is used for obtaining motion information. The design of this hybrid camera is the equivalent to the used PET/CT scanner. PET is in that case the low spatial resolution detector and the CT is the high spatial resolution one. The 4D-CT provided the frames, like the secondary detector in [24]. The most different part is, that PET/CT data is a complete 3D data set, the hybrid system in [24] only provided 2D images.

Therefore, we implemented full 3D deconvolution of PET images with a PSF created by 4D-CT. In the proposed algorithm no changes in patient setting or acquisition protocol of the PET data have to be done. Therefore, the algorithm is also independent of the hardware specifications of the scanner and can even be applied retrospectively to PET data as long as a 4D-CT or any motion information is available for this patient.

### Motion estimation and PSF modeling

The next essential step after acquiring the data is the estimation of the individual motion and the motion modelling for correction. Naqa et al. [25] used a deblurring technique that combines patient-specific motion estimates of tissue trajectories with image deconvolution techniques. The model they used to estimate the motion trajectories was derived from a linear mapping of the tidal volume/airflow space into 3D space at different phases of the breathing cycle [26]. The model accounts for the observed hysteresis-like behavior of the lung motion [14]. We used a similar approach to correct 3D-PET data for motion, especially for respiratory motion. While Naqa et al [25] just applied a 2D transformation/correction to each 3D image slice we modeled a complete 3D correction, using the binned and sorted 3D data sets from the 4D-CT.

The end-inspiration and end-expiration CT data sets were obtained from the 4D-CT and movement between these two phases was detected using a commercial rigid registration algorithm included in the Mediso Interview Fusion software (Mediso Medical Imaging Systems Ltd., Budapest, Hungary). As optimal motion correction of the tumor was the primary goal, for registration a bounding box around the tumor was drawn for optimal co-registration in this area. The motion vector was used as motion trajectory which can be transferred in a 3D PSF representing the whole motion.

### Deconvolution and image restoration

Naqa et al [26] found out, that for deconvolution in clinical PET data, blind deconvolution is not suitable. They estimated in their approach the breathing motion model from 4D CT measurements, based on [26–28].

We used the iterative Richardson-Lucy-Deconvolution-Algorithm using this CT-registration based PSF for deconvolution of the 3D-PET images:

$$corr_{k+1}\left(x,y,z\right)=corr_{k}\left(x,y,z\right)\left[PSF\left(-x,-y,-z\right)⊗\left(\frac{PET\left(x,y,z\right)}{PSF\left(x,y,z\right)⊗corr_{k}\left(x,y,z\right)}\right)\right]$$

$corr{\;}_{k}\left(x,y,z\right):$Motion corrected 3D-PET image after k-th iteration step, PSF: Point Spread Function, PET: blurred PET image. Multiplication and division are point-wise.

We assume the PSF to be constant (spatially invariant) over the whole image. Therefore, rigid registration between each CT was used to obtain just one motion vector for the whole dataset.

As starting image there are two different options available. First, we used an image which all pixels had the same constant value $\frac{1}{number\;of\;pixels}$. Second, we used the 3D blurred PET image.

The algorithm was applied to phantom data using these two options of the starting image. Consequently for phantom data different iteration number (n=1 to 9) were used in the algorithm to assess the optimal number of iterations.

Finally, the correction algorithm was applied to 3 patients’ data sets using the parameters which have been found to be optimal in phantom studies. For patient studies two corrections were performed, 1^st^ just in cranio-caudal direction with a linear PSF in this direction and 2^nd^ the correction for which the PSF is based on the full-3D-trajectory estimated previously.

### Motion phantom measurement and image analysis

**Figure 4:**
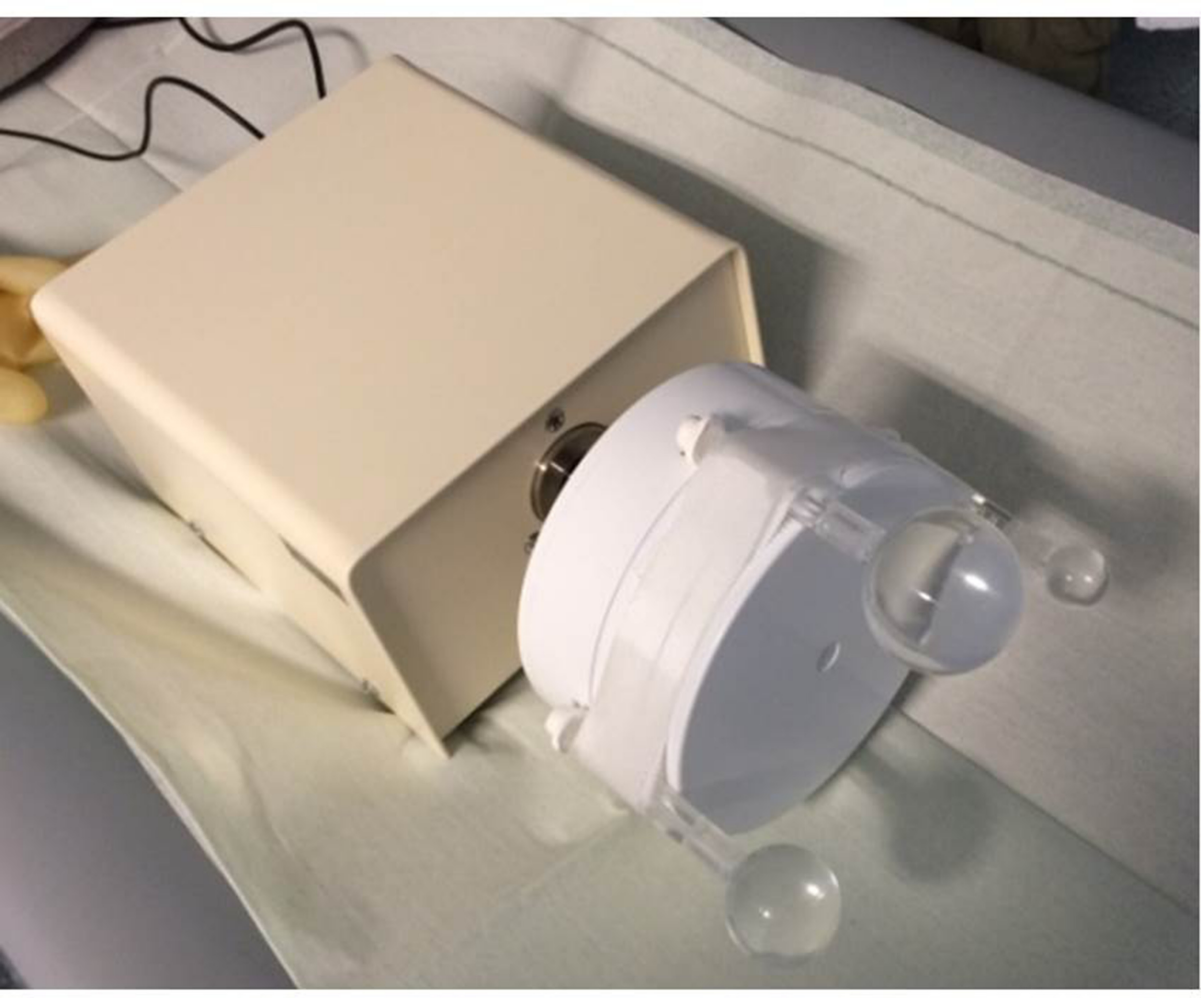
Motion phantom with the three attached filled spheres A systematic analysis was done to optimize the parameters (number of iteration, start image) of the deconvolution.

To show the feasibility of the developed algorithm, PET/CT-motion phantom measurements were performed using an extended CT-motion phantom, which is provided in the basic form without the fillable sphere attachments by Anzai (Anzai Medical CO, LTD, Tokyo, Japan) (Figure 4). The phantom consists of two parts, the body with motor and a moving cylinder. The phantom can be connected to the pressure sensor, which is normally placed in an elastic belt around the patient’s thorax to detect the respiratory movement. We extended this phantom for using it in CT and PET and attached 3 fillable spheres (3, 12, and 26 ml volume, activity concentration 1.2 MBq/ml) simulating tumor lesions moving periodically and smeared over a larger volume with a maximal movement of 15 mm (Figure 1). Data of the moving phantom were acquired 4 minutes for one bed position centered on the moving lesions. For comparison a static PET image of the activity filled spheres was acquired as well for 4 minutes. All phantom PET images were reconstructed into a 128 x 128 matrix using the iterative reconstruction algorithm (4 iterations, 8 subsets). Volume and activity concentration were measured for comparison of the spheres in PET images. For volume measurements an isocontour with 40% of the maximum threshold was used. The max-activity concentration was measured with this volume. The same methods have been applied to analyze data of the 3 patients that haven been obtained FDG-PETC/T scans before planned stereotactic body radiation treatment. All three patients had solitary, biopsy-proven non-small-cell lung carcinoma and were treated in a curative attempt, more details about each patient can be found in Table 2. All patients gave written and informed consent for the diagnostic procedure. Due to the retrospective character of the patient data evaluation ethical commitment was waived by the institutional ethics board. For patient imaging tumor volume, maximum and mean activity uptake of the lesions were compared before and applying the two correction methods.

**Table 2:** Patient data

	Age	Gender	Patient weight	Tumor location
Patient 1	66 years	male	80 kg	right middle lobe
Patient 2	55 years	male	78 kg	left lower lobe
Patient 3	67 years	male	81 kg	right lower lobe

## Abbreviations

CT: computed tomography
EM: expectation maximization method
PET: positron emission tomography
PSF: point spread function
